# A Journey toward Immediate Denture: Overcoming Bitter Reality with Psychotherapy

**DOI:** 10.1155/2022/3080039

**Published:** 2022-07-26

**Authors:** R. Varsha, P. S. Manoharan, Vigneshvar Chandrasekaran, K. Savitha

**Affiliations:** ^1^Department of Prosthodontics, Indira Gandhi Institute of Dental Sciences, Sri Balaji Vidyapeeth Deemed-to-Be University, Pillaiyarkuppam, Puducherry 607 402, India; ^2^Department of Psychiatry, Mahatma Gandhi Medical College and Research Institute, Sri Balaji Vidyapeeth, Deemed-to-Be University, Pillaiyarkuppam, Puducherry 607 402, India; ^3^Department of Prosthodontics, Mahatma Gandhi Postgraduate Institute of Dental Sciences, Kalaivaanar Nagar, Puducherry 605006, India

## Abstract

The loss of teeth affects the aesthetics, function (mastication and speech), confidence, mental state, and the overall quality of life of an individual. Aggressive periodontitis is a destructive condition leading to loss of teeth at early stages of the disease. Individuals facing this inevitable condition of losing the teeth and replacement at a younger age experience formidable psychological distress. The prosthodontic procedure when supported with psychotherapeutic interventions can support the patient in accepting the prognosis and treatment. We report the scenario where psychological intervention was provided in a systematic manner adopting the SPIKES protocol for a 42-year-old man presenting with aggressive periodontitis.

## 1. Introduction

The loss of teeth, apart from affecting the structural integrity, nutrition, mastication, and taste perception associated with the functions of teeth, also affects the emotional stability, self-image, confidence leading to disturbances in physical and overall well-being of an individual [1].

Aggressive periodontitis is a condition leading to early tooth loss. It is characterized by rapid loss of periodontal ligament and the alveolar and the interproximal attachment loss that affects more than three teeth during or even before the onset of puberty. Furthermore, the symptoms of mobility are noticed quite late when the destruction of teeth have progressed to a higher severity. In severe presentations of the disease, individuals present with mobile teeth and an inability to eat. In such individuals, the prognosis of the condition is considered very poor, leaving the dentist with fewer options apart from the definitive solution of removing all the teeth with prosthetic rehabilitation [2]. Aggressive periodontitis can be managed by mechanical debridement accompanied by antimicrobial agents, scaling, and root planing effectively; however, it may not ensure long term stability [3, 4].

Due to the unawareness of the aggressive nature of the disease, the patients' acceptance of the prognosis and the rehabilitation measures are limited. The provision of a systematic psychological intervention for “breaking the bad news” can lead to improved acceptance and outcomes in such individuals [5]. It is expected of a dentist to understand, diagnose, and carefully plan the treatment considering the benefits of psychological interventions [6]. The literature opines that training of undergraduate and postgraduate should encompass a well-structured training module for providing the appropriate knowledge, attitude, and communication skills, which are needed to counsel patients [7]. In this report, we discuss about a middle aged male with aggressive periodontitis who was provided psychological intervention as a part of management and rehabilitation.

### 1.1. Case Presentation

**Figure 1:**
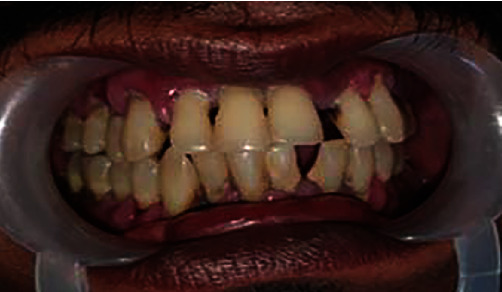
Clinical oral examination.

**Figure 2:**
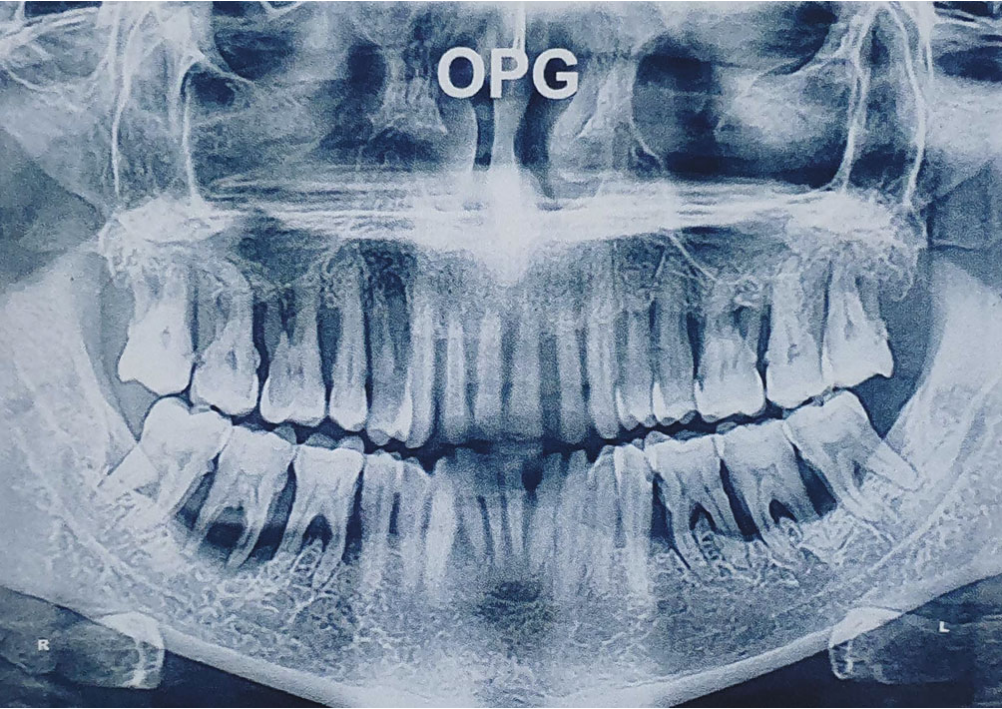
Orthopantomograph examination.

A 42-year-old male patient reported to the Department of Prosthodontics, complaining of severely mobile maxillary and mandibular teeth and the inability to chew with the existing dentition. The patient had been diagnosed of diabetes and was on regular medications for past 5 years. On examination the patient presented with Grade II mobility in the anterior and Grade III mobility and Grade II to III gingival recession and furcation involvement (Figure 1). The examination findings were explained to the patient and was emphasized the need for further investigations. Orthopantomograph revealed generalized severe vertical bone loss around all the teeth (Figure 2). The prognosis of all the remaining natural teeth was hopeless as per the opinion of the periodontist.

A diagnosis of aggressive periodontitis was made according to the guidelines presented by AAP [1999] classification by the periodontists and reported that all the teeth had hopeless prognosis, with the advanced bone loss and clinical attachment loss in all the teeth. Total extraction and replacement of teeth was considered as the only option. The patient owns a business where he would have to face customers on a daily basis due to which the impending loss of teeth would be quite distressful and unacceptable for the patient.

The bad news was communicated using the SPIKES protocol [8] as follows (Table 1).

All the possible pros and cons for each treatment option was explained in simple words. Educational models and videos were used to explain all the treatment options. The patient was given ample opportunity to ask questions and express his limitations in terms of affordability and time. The patient opted for immediate denture based on the factor that he could not be without teeth owing to his profession and affordability. After the patient chose the option, photographs of other patients with a similar condition were shown for better appreciation of the course of treatment. The need for relining within the first year was also explained to the patient [9].

**Figure 3:**
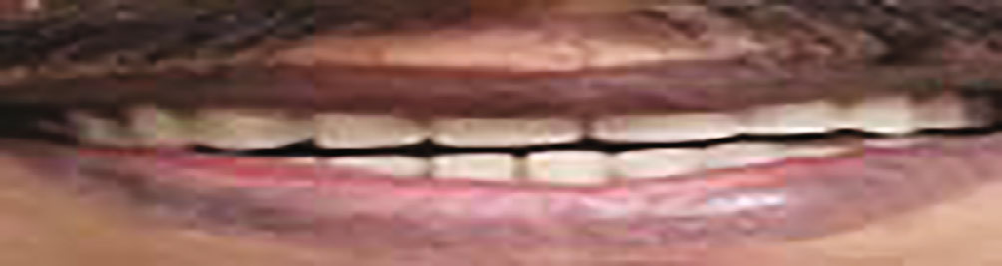
Oral examination after Immediate denture replacement (after 3 months).

The treatment plan for a conventional immediate denture was adopted [10]. The patient was reviewed after 3 months (Figure 3). The patient had performed well with the dentures with respect to day-to-day functions of mastication and speech. The patient also mentioned that he would consider replacement with implants as an option at a later date [11]. The OHIP EDENT questionnaire was used to assess the patient before and after the usage of dentures, and it was found that the oral health quality of life had improved for the patient after the usage of dentures.

## 2. Discussion

The level of emotional distress, loss of confidence, and self-image is often overlooked during the treatment of aggressive periodontitis [12]. The wholesome management including psychotherapy of such patients would help the patient to go through a smooth transition from a dentate to an edentate individual with the prosthesis [13].

In the literature pertaining to dentistry, breaking the bad news involves informing about a suspicious lesion, information on loss of teeth, information of a surgical procedure or a procedure with poor prognosis, or the information of a lesion that reflects a likely systemic condition. Other models of breaking bad news such as ABCDE model, PACIENTE protocol, and BREAKS protocol have been mentioned to be used as guidelines for breaking the bad news [14] (Table 2).

In this case report, we have adopted the SPIKES protocol in systematically educating and motivating the patient toward accepting his disability, the poor prognosis, and possible treatment options [8]. The patient gained confidence and assurance from the operator and was quite ready when the last day of extraction of anterior teeth were planned. The patient understood the need for future procedures such as reline and accepted to undergo other further definitive options at a later date.

Tooth loss has a significant impact on the emotional well-being of the patient and should be handled in a systematic and scientific manner. Adequate use of guidelines for breaking bad news by trained personnel would alleviate the apprehension and the associated anxiety and distress ultimately leading to acceptance of prosthodontic rehabilitation.

## Figures and Tables

**Table 1:** The SPIKES protocol.

Setting ambience and privacy	A private counseling set up was planned for the patient along with his spouse without any external interruptions.
Perception (how much the patient knows)	He was totally unaware of the condition and may never accept the aforementioned treatment.
Invitation or information	The patient was explained about the situation with all the supporting radiographs and diagnostic casts on the severity of the condition.
Knowledge sharing	Simple words were used and practical issues related to understanding of the disease was conveyed gradually in a piece meal approach.
Empathy	The anxiety of the patient and the bystander was very evident from their body language as the severity of the problem was revealed to its fullest. We felt a stoic silence and they were trying to conceal disappointment and emotional distress. We asked them to express what they felt at that moment. We did not get any response or reply but a silent pause and an anticipation of some hope.
Summarize	At that point we decided to disclose to the patient that, it was very unfortunate that his remaining teeth had a very hopeless prognosis. The patient was also explained that retaining his remaining teeth will in no manner alleviate the present situation.
Strategise	Although the patient and the relatives came to terms with the reality, they wanted to know possible options for an alternative. The plan of sequential extraction and immediate denture was explained to the patient. The patient was given four different treatment options based on the diagnostic work up.

**Table 2:** Models of breaking bad news.

Model	Characteristics
ABCDE model	A—Advance preparation B—Build a therapeutic environment/relationship C—Communicate well D—Deal with patient and family reactions E—Encourage and validate emotions (reflect back emotions) [15]
PACIENTE protocol	P—Prepare A—Assess how much the patient knows and how much they want to know C—(*Convite à verdade*, in Portuguese) Invite the patient to the truth I—Inform E—Emotions N—(*Não abandone o paciente*, in Portuguese) Do not abandon the patient TE—(*trace uma estratégia*, in Portuguese) Outline a strategy P-A-C-I-E-N-T-E stands for (“patient,” in Portuguese) [16]
BREAKS protocol	B—Background R—Rapport E—Explore A—Announce K—Kindling S—Summarize [17]
